# Menstrual hygiene management practices among rural females: findings from a rural health demographic environmental surveillance system (HDESS) cohort in Odisha, Eastern India

**DOI:** 10.3389/fgwh.2025.1617970

**Published:** 2025-10-13

**Authors:** Swetalina Nayak, Mamata Nayak, Soumya Ranjan Nayak, Debasini Parida, Bijaya Kumar Mishra, Abhinav Sinha, Sanghamitra Pati, Gayathri Delanerolle, Peter Phiri, Ashish Shetty, Sohier Elneil, Biswabara Rout, Jyotirmayee Turuk, Subrata Kumar Palo

**Affiliations:** 1 Department of Public Health, ICMR- Regional Medical Research Center, Bhubaneswar, India; 2 Department of Public Health, University College London Hospitals NHS Foundation Trust and University College London, London, United Kingdom; 3 Department of Public Health, SCB Medical College, Cuttack, Odisha, India; 4 Department of Public Health, ICMR- Regional Medical Research Center, Bhubaneswar, Odisha, India

**Keywords:** menstrual hygiene management, rural area, sanitary pads and disposal method, students, community women

## Abstract

**Introduction:**

Menstrual hygiene management is an important public health component to promote reproductive health, especially in rural areas of low-and middle-income countries (LMICs). Present study assessed the menstrual hygiene practices, the disposal methods and associated health effects among **school students, college students, and community women** in rural setting of a Health Demographic Environmental Surveillance System (HDESS) in Odisha, India.

**Methods:**

A cross-sectional survey was carried out in March-May 2023 using three cohorts of participants, namely school students, college students, and community women aged between 14 and 49 years and currently experiencing cycles of menstruation. The study used a multi-stage random sampling method with a descriptive analysis comparing patterns of sanitary pad use, its disposal practice and complications linked to the use of pads. Continuous variables such as age and cost incurred on menstrual products were reported using mean and standard deviation. The chi-squared value was used to determine possible associations and affirm the statistical significance, *p*-value (<0.05).

**Results:**

Among 622 participants, 541 (86.9%) reported using sanitary pads. Cloth users rated their experience as “excellent” (48.7%), while dissatisfaction was highest among government-provided pad users (29.7%). Self-procured pad users reported the highest “manageable” experience (44.9%). The most common method of disposing the sanitary materials was by burying (40.2%). Approximately 68.32% of participants reported menstruation related health issues whilst 23.9% reported sanitary pad-related issues. About 16.8% of the participants reported of menstrual irregularities, school students being the most (44.8%).

**Discussion:**

The use of disposable sanitary pads appear to be the most common menstrual hygiene management practice in rural areas. Awareness, shame, and costs could be barriers to accessing sanitary pads along with poor environmental impact when disposing of these using improper methods. Reform the policy by introducing awareness of overall health implications linked to menstrual hygiene and access to low-cost menstrual management products could promote reproductive health. Also, government partnerships with NGOs to implement village-level menstrual waste management systems, subsidies for eco-friendly pad production, and mandatory disposal education in school health programs could mitigate the problem.

## 1 Introduction

Menstruation is a key aspect of women's reproductive health and is experienced by nearly 1.9 billion women aged 14–49 worldwide (1). However, in rural India, challenges persist in menstrual hygiene management (MHM) due to limited access to menstrual products, inadequate awareness, and social taboos. MHM—defined as the use of clean materials to absorb menstrual blood, changed in privacy as needed, with access to soap, water, and safe disposal (2)—remains understudied in rural populations, particularly regarding practices, product satisfaction, and health outcomes.

Globally, menstrual products range from homemade materials (e.g., cloth, cotton) to commercial options (e.g., sanitary pads, menstrual cups) (3). In rural India, 64% of women still rely on cloth due to affordability and accessibility barriers (4), despite risks of infections (UTIs, bacterial vaginosis) from improper use (5, 6). To address these gaps, several government initiatives have been launched. The Menstrual Hygiene Scheme (MHS) under the Adolescent Reproductive and Sexual Health (ARSH) program and the Khusi scheme in Odisha aim to increase awareness, accessibility, and affordability of sanitary pads by providing subsidized packs to adolescent girls in rural areas (7, 8). These programs also emphasize safe disposal, which remains a major environmental and public health concern.

Despite these efforts, gaps still exist in product accessibility, usage satisfaction related to the types of pad, and awareness— with limited data comparing demographics. For instance, school students may lack autonomy in product choice, college students might have better awareness due to higher education, and community women face distinct socioeconomic constraints. Furthermore, much of the existing research has focused on urban settings, creating a scarcity of data that truly reflects the realities of rural areas (9). Moreover, the extent to which government-supplied sanitary products meet the needs and expectations of users in different demographic groups is still unclear.

Therefore, the present study was conducted among three distinct groups—school-going students, college students, and community women—in a rural Health and Demographic Surveillance System (HDSS) area. This grouping was intended to reflect different stages of life, education, and access to information and resources. The study aimed to explore current menstrual hygiene practices, assess satisfaction with the types of menstrual materials used, identify any associated health problems, and understand disposal practices. These aspects are crucial for evaluating the effectiveness of existing menstrual health initiatives and identifying areas that need targeted interventions.

## 2 Materials and methods

### 2.1 Study design and study setting

A cross-sectional survey was carried out during March-August 2023 at model rural health research unit (MRHRU) located at Tigiria, Odisha, India. The participants were from an rural Health Demographic Environmental Surveillance System (HDESS) established under the unit (MRHRU). The HDESS comprises of a total population size of 76,379 from 48 rural villages and of which 20,837 (27.3%) are female in the age group 14–49 years (10).

### 2.2 Study participants

Under the study, a total of 622 eligible participants from three different categories a. school students (206 nos.), b. College students (206 nos.), and c. women from the community (210 nos.) were enrolled. Participants in the reproductive age group (14–49 years), experiencing menstrual cycles and agreed to provide written informed consent were included. Pregnant women, lactating mothers(<6 months of child birth), immobile, and those with recognizable cognitive impairments were excluded.

### 2.3 Sample size calculation

Considering that 57% of women of reproductive age in India use sanitary pads (11) and with an estimated absolute precision of 5.7% (relative precision as 10% of prevalence) was considered to a heterogeneity of 1.5, with a non-response rate of 10%, the desired sample size was estimated to be 530. Finally, a total of 622 participants were enrolled taking into account ±1% error rate.

The following formula was used to estimate the desired sample size:

N=[deff*Np(1-p)]/[(d2/Z21-α/2*(N-1)+p*(1-p)

where, *n* = sample size, Deff = design effect, *N* = population size, p = estimated proportion, q = 1-p, d = desired precision or absolute level of precision.

### 2.4 Sampling method

**Figure 1:**
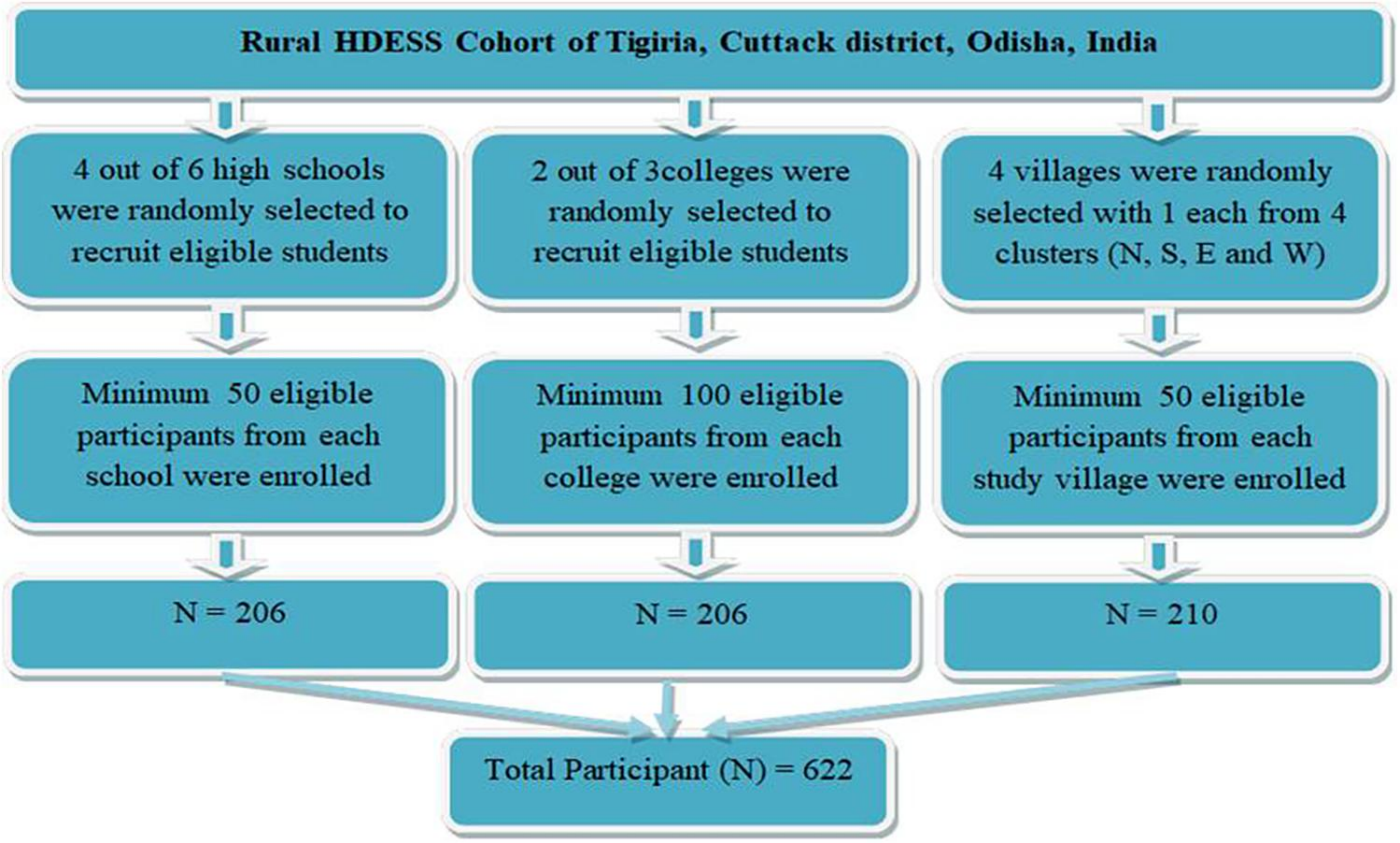
Process followed for participant recruitment.

**Figure 2:**
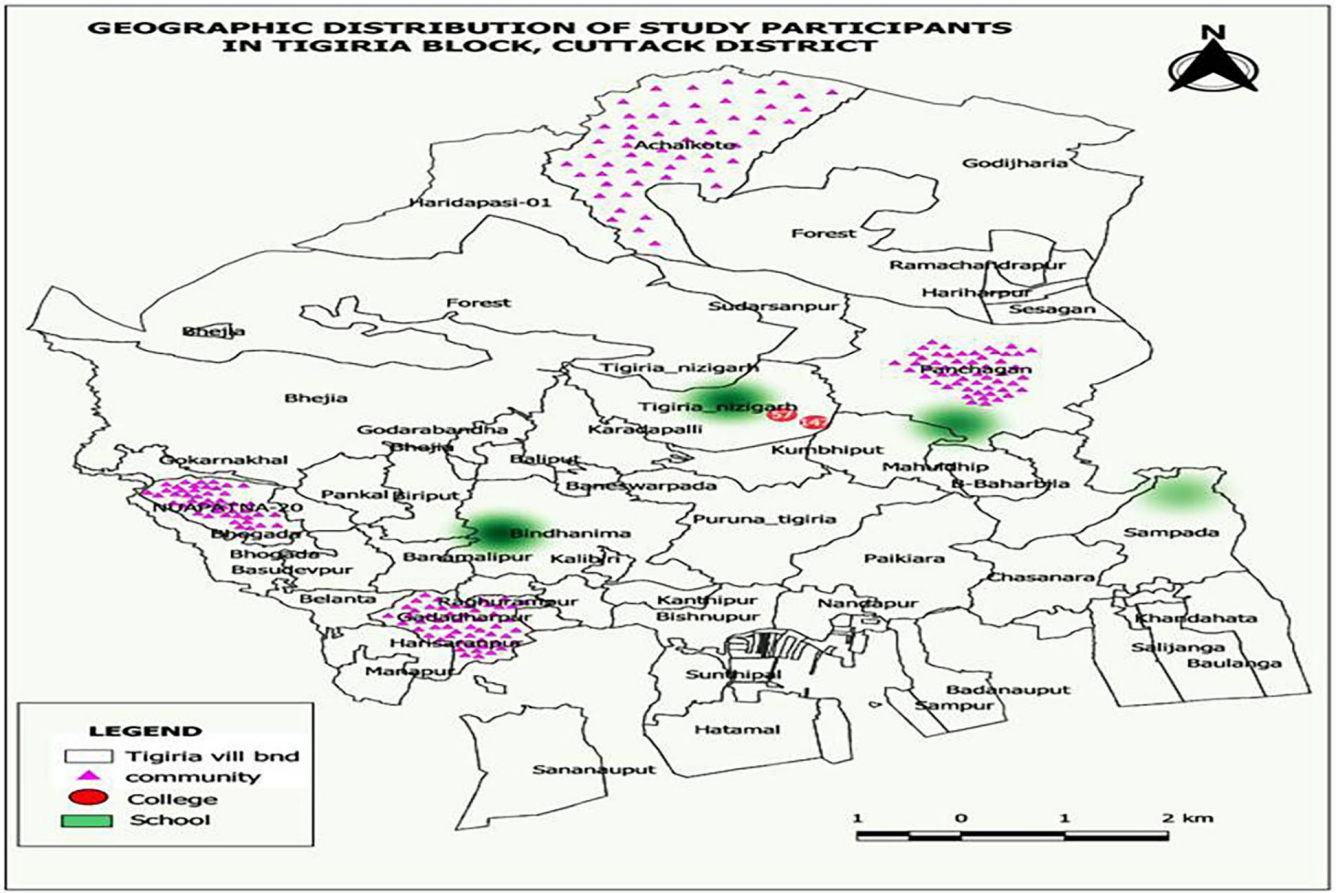
Heat Map showing geographical distribution of the study participants data collection.

We attempted to recruit 200 participants from each study group for equitable representation across the three cohort. This ensured feasibility while maintaining comparative analysis power across subgroups and avoid overrepresentation of any single group. Four schools and two colleges were randomly selected out of six schools and three colleges, respectively (available in the study setting). Four villages were randomly selected with one each from each of the directional-clusters of North, South, East, and West for better geographical representation. A minimum of 50 community women participants were recruited from each of the selected villages. Survey team visited consecutive households (following HDESS survey method) in the villages until the desired sample size is acheived. In case of non-availability of any eligible participant, the neighbour household was considered. The process followed in recruiting the study participants and their geographic distribution in the HDESS area are presented in Figures 1, 2 respectively.

Data were collected from the study participants using a validated questionnaire**.** The questionnaire was adapted from prior menstrual hygiene studies and validated through expert review (5 public health/gynaecology experts) and pilot-testing (30 participants). Some modifications were made for cultural appropriateness in rural settings.

Information pertaining to socio-demographic characteristics, menstrual experiences and hygiene management were collected. Data was collated in a pre-defined study specific Excel template and exported to STATA software for analysis.

### 2.5 Variable description

This study included socio-demographic information like age groups (less than 20, 20–29 years, 30–39 years and 40 and above), years of schooling (<5 years complete, 5–15 years of complete, 16–22 years of complete), family income (<Rs.10000, Rs.10000–24999, >Rs. 25000), types of pad use (sanitary pads including government and self procured pad and clothes including handmade, cotton and gauges), disposal methods (throwing outside, burning and burying). Additionally, it included menstrual history like regularity of cycles (We defined an irregular menstrual cycle if the gap between two cycles were shorter than 22 days or more than 41 days) (12), problems during menstruation(self reported), years since menarche and average duration of menstruation, average pad requirement per cycle and average cost incurred per cycle. In problem during menstruation, problems like abdominal pain, back pain, head ache, Hand and leg pain, weakness, nausea, vomiting are repotrd and are completely self reported. Among problems related to sanitary pad use included Itching, skin rashes, and ittitation and is also self reported.

### 2.6 Data analysis

The statistical analysis was completed using STATA v.17.0 (Stata Corp, Texas) software. Descriptive statistical approaches were used to determine the socio-demographic and menstrual history profile of the participants and presented as frequency and proportion with their 95% CI. Mean and standard deviations were calculated for continuous variables. The chi-square test was used to determine the association between types of the pad with various independent characteristics and the *p*-value (<0.05) was considered as significant. We addressed potential confounding through stratified analyses by key demographic variables (age, education, income) where feasible. For example, satisfaction levels with pad use were compared across subgroups (students vs. community women) to assess consistency. The types of health problems due to the use of sanitary pads and their disposal methods are depicted graphically. In case of missing values, we tried to recollect the data through repeated visisting the site. Hence, there was very few missing data that we excluded during data analysis.

### 2.7 Ethical considerations

Ethical approval was obtained from the institutional ethics committee of the ICMR-Regional Medical Research Center, Bhubaneswar (vide reference no: ICMR-RMRC/IHEC-2022/103). Informed written consent was obtained from all the study participants and written assent was obtained from the student participants who were under 18 years. Confidentiality and anonymity were maintained throughout the study and data security was ensured.

## 3 Results

### 3.1 Demographic characteristic and menstrual history of participants

Among the 622 study participants, 66.2% were students—equally divided between school (33.1%) and college students (33.1%)—while the remaining 33.8% were community women. Approximately, 7.14% of the community women were employed. The mean age of the participants was 20.9 (±8.2) years.

The average duration of menstruation was 4.84 ± 1.26 days and was longer among school students (5.36 ± 1.16 days). Overall, 16.88% of the participants reported menstrual irregularities, with 44.8% being school students, followed by women from the community (34.3%). The detailed socio-demographic characteristics and menstrual history along with their 95% confidence interval are presented in Table 1.

**Table 1:** Socio-demographic characteristics and menstrual history of study participants (*N* = 622).

Variables	School student (*N* = 206)	95% CI	College student (*N* = 206)	95% CI	Community women (*n* = 210)	95% CI	Total (*n* = 622)
Age group							
Less than 20 years	206 (49.8)	–	195 (47.1)	(42.2-52.03)	13 (3.1)	(1.6–5.3)	414 (66.55)
20–29 years	–	–	11 (10.8)	(5.5–18.4)	91 (89.2)	(81.5–94.4)	102 (16.39)
30–39 years	–	–	–	–	71 (100)	(94.9–100*)	71 (11.41)
40 years and above	–	–	–	–	35 (100)	(89.9–100*)	35 (5.62)
Total	206 (33.1)	–	206 (33.1)	–	210 (33.8)	–	622
Years of schooling							
<5 years complete	–	–	–	–	10 (100)	(0.69–1*)	10 (1.60)
5–15 years of complete	206 (54.5)	(49.3–59.5)	2 (0.5)	(0.06–1.8)	170 (45)	(39.8–50.13)	378 (60.77)
16–22 years of complete	–	–	204 (87.2)	(82.20–91.18)	30 (12.8)	(8.8–17.7)	234 (37.62)
Total	206 (33.1)	–	206 (33.1)	–	210 (33.8)	–	622
Types of pad							
Sanitary pad (govt.pad/self procured)	205 (37.8)	(33.78–42.13)	205 (37.8)	(33.78–42.13)	131 (24.21)	(20.6–28)	541 (86.97)
Clothes/handmade/cotton gauge	1 (1.23)	(0.03–6.6)	1 (1.23)	(0.03–6.6)	79 (97.53)	(91.3–99.6)	81 (13.02)
Total	206 (33.1)	–	206 (33.1)	–	210 (33.7)	–	622
Disposal method (*N* = 541)							
Burning	6 (23.08)	(8.9–43.6)	4 (15.38)	(4.3–34.8)	16 (61.54)	(40.5–79.7)	26 (4.18)
Burring	80 (32)	(26.2–38.1)	109 (43.6)	(37.3–49.9)	61 (24.4)	(19.2–30.2)	250 (40.25)
Throwing outside	54 (30.51)	(23.82–37.8)	44 (24.86)	(18.6–31.8)	79 (44.63)	(37.1–52.2)	177 (28.50)
Others	66 (39.29)	(31.8–47.1)	49 (29.17)	(22.4–36.6)	53 (31.55)	(24.6–39.1)	168 (27.05)
Total	206 (33.17) ()	–	206 (33.17)	–	209 (33.66)	–	621
Family income (monthly)							
Below 10,000	98 (36.0)	(30.3–42)	68 (25.0)	(19.9–30.5)	106 (39.0)	(33.1–45)	272 (43.72)
10,000–24,999	82 (28.8)	(23.58–34.4)	115 (40.3)	(34.6–46.2)	88 (30.9)	(25.56–36.5)	285 (45.81)
25,000 and above	26 (40)	(28–52.9)	23 (35.4)	(23.9–48.2)	16 (24.6)	(14.7–36.8)	65 (10.45)
Total	206 (33.1)	–	206 (33.1)	–	210 (33.7)	–	622
Menstrual cycle-related determinant							
Regularity of cycles							
Regular	159 (30.7)	(26.7–34.9)	184 (35.6)	(31.4–39.8)	174 (33.7)	(29.5–37.9)	517 (83.11%)
Irregular	47 (44.8)	(35.04–54.7)	22 (20.9)	(13.6–29.9)	36 (34.3)	(25.2–44.1)	105 (16.88%)
Total	206 (33.1)	–	206 (33.1)	–	210 (33.7)	–	622
Problems during Menstruation							
No	40 (20.3)	(14.9–26.6)	42 (21.3)	(15.81–27.7)	115 (58.4)	(51.1–65.3)	197 (31.6)
Yes	166 (39.1)	(34.3–43.8)	164 (38.6)	(33.9–43.3)	95 (22.3)	(18.4–26.6)	425 (68.32)
Total	206 (33.1)	–	206 (33.1)	–	206 (33.8)	–	622
Years since menarche	1.76 ± 1.09	1.67–1.84	4.78 ± 1.20	(4.68–4.87)	16.55 ± 6.68	(16.02–17.07)	7.75 ± 7.54
Average duration of menstruation (in days)	5.36 ± 1.16	5.26–3.45	4.83 ± 1.17	(4.73–4.92)	4.38 ± 1.25	(4.28–4.47)	4.84 ± 1.26
Average pad requirement per cycle	11.67 ± 3.96	11.32–12.01	9.69 ± 3.27	(9.40–9.97)	9.51 ± 3.97	(9.16–9.85)	10.4 ± 3.84
Average cost incurred per cycle (Rs.)	45.64 ± 20.05	43.87–47.40	53.20 ± 28.13	(50.72–55.67)	43.27 ± 20.73	(41.44–45.09)	48.12 ± 24.20

### 3.2 Use of sanitary materials and the cost incurred

Of the total sample, 541 (86.9%) used sanitary pads—either the purchased ones or the government-supplied—whilst the remaining 81 (13.1%) used old clothes or handmade pads. Among the cloth users, 97.5% were women from the community and 2.4% were school students. None of the participants were found to use other sanitary products such as menstrual cups, tampons, or discs.

The average cost incurred to manage menstrual flow was found to be 48.12 (±24.20) Indian rupees (INR) per cycle, which was similar across all three categories of study participants.

### 3.3 Problems related to menstruation and material used

Menstruation-related health problems such as body pain, abdominal pain, skin rashes, heavy bleeding, and headaches were reported by 68.32% of participants (39.1% among school students and 38.6% among college students).

### 3.4 Level of satisfaction, requirement, and disposal methods related to use of sanitary material

Among the participants using sanitary pads, 69.5% used only self-procured sanitary pads, 8.7% used only government-supplied pads and 21.8% used both. Approximately 91.5% of those using government-supplied pads were school students and 8.5% were college students. Among the self-procured participants, 52.7% were college students, 34.8% were community women, and 12.5% were school students (*P* value < 0.05).

The satisfaction level with using sanitary pads was assessed using a 4-pointer Likert scale (excellent, satisfactory, manageable, and dissatisfied). Satisfaction levels varied significantly based on the type of sanitary material used (*χ*^2^ = 148.45, *p* < 0.05). Cloth users reported the highest satisfaction, with 48.7% rating their experience as “excellent.” In contrast, 29.7% of government pad users rated their experience as “dissatisfied”. Self-procured pads were most commonly rated as “satisfactory” (44.9%) followed by “manageable” (32.1).

Among the participants, disposal methods showed considerable variation. Burying used pads was the most common method (40.2%), followed by discarding them in open areas (throwing outside) (28.5%) and other methods (27.05%) that included throwing in latrines, running water, ponds, and rivers (*P* value < 0.05). Burning was the least practiced method (4.1%), though it was more prevalent among cloth users (16.05%) (*P* value < 0.05).

Among the sanitary pad users, 149 (23.9%) reported pad-associated problems of whom 4 are refused to give their data. Amomg the participants who mentioned their problems, 44.6% among users of government-supplied pads, 19.1% among self-procured users, 46.6% among those using both, and 1.12% among cloth users. The associated health problems were mostly itching (70.4%), irritation (17.2%), and skin rashes (12.4%).

**Figure 3:**
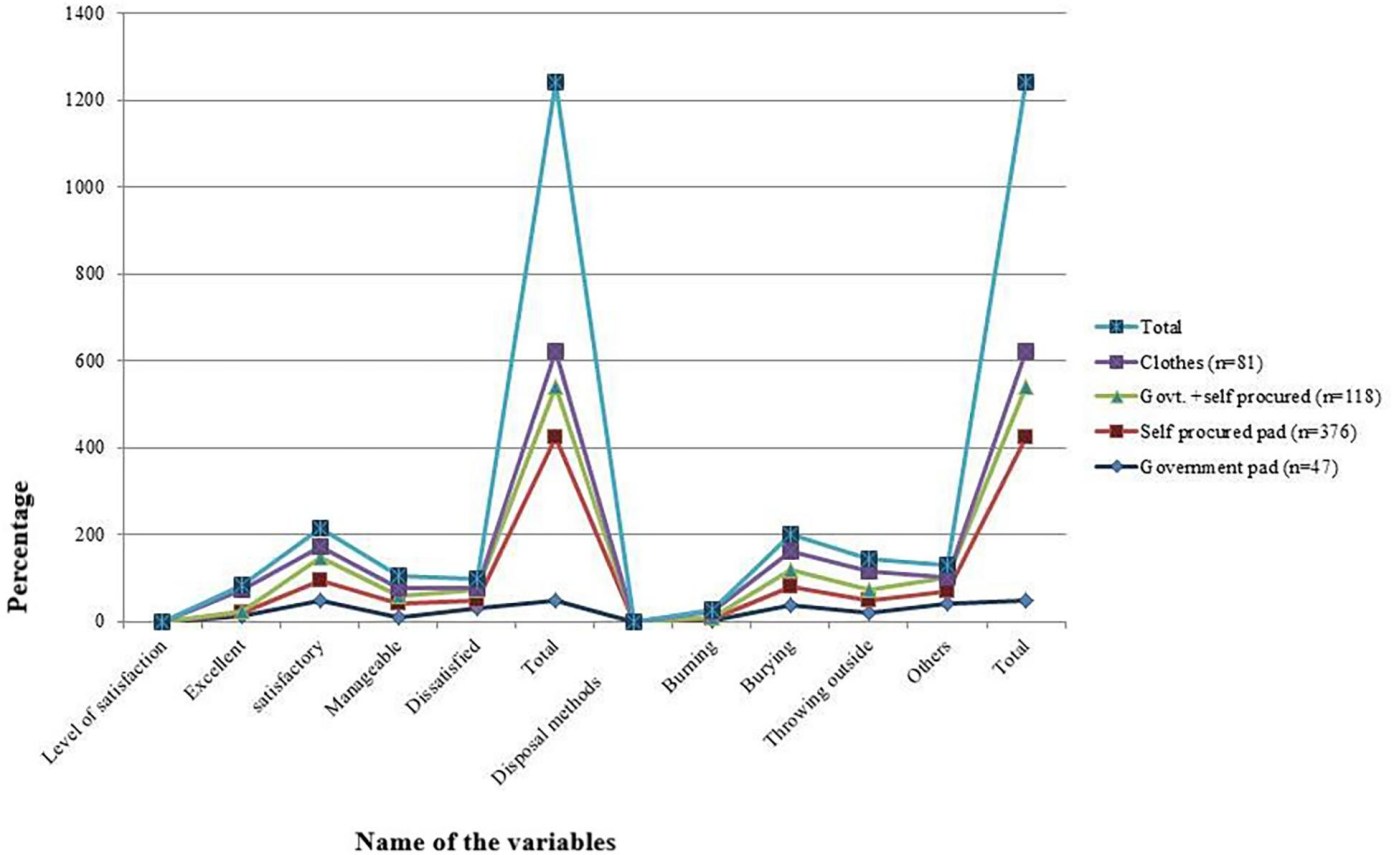
Problems due to the use of sanitary pads among different categories of study participants.

We also estimated the cost incurred among different pad users. Whereas the average cost incurred per cycle among sanitary pad users was INR 48.12 (±24.20), it was INR 48.91 (±24.72) among self-procured users, INR 26.67 (±5.77) among users of government-supplied pads, and INR 46.13 (±22.50) among users of both. The differences among the different types of pad users are detailed in Table 2. Additionally, the differences in cost and the requirement of pads (in numbers) are appended in Supplementary Table S1. The pads with different types, their disposal methods and level of satisfaction is illustrated in Figure 3.

**Table 2:** Level of satisfaction, disposal methods, requirements, and problems related to types of sanitary pads.

Name of the variables	Government pad (*n* = 47)	Self procured pad (*n* = 376)	Govt. +self procured (*n* = 118)	Clothes (*n* = 81)	Total	Chi-square value, *P*-value
Level of satisfaction						
Excellent	6 (12.8)	20 (5.3)	7 (5.9)	39 (48.8)	72 (11.6)	chi2 = 148.4521, Pr = 0.000
satisfactory	23 (48.9)	169 (44.9)	62 (52.6)	20 (25)	274 (44.1)	
Manageable	4 (8.5)	121 (32.2)	20 (16.9)	16 (20)	161 (25.9)	
Dissatisfied	14 (29.8)	66 (17.6)	29 (24.6)	5 (6.2)	114 (18.4)	
Total	47	376	118	80	621	
Disposal methods						
Burning	1 (2.1)	8 (2.1)	4 (3.4)	13 (16)	26 (4.2)	chi2 (9) = 65.6883 Pr = 0.000
Burying	18 (38.3)	152 (40.5)	46 (39.0)	34 (42)	250 (40.3)	
Throwing outside	9 (19.1)	103 (27.5)	31 (26.3)	34 (42)	177 (28.5)	
Others	19 (40.5)	112 (29.9)	37 (31.3)	0	168 (27.0)	
Total	47	375	118	81	621	
Requirement per day						
1–2 pads	33 (70.2)	274 (73.1)	83 (70.4)	–	391 (72.2)	Chi2 (6) = 1.3360 Pr = 0.970
3–4 pads	13 (27.7)	97 (25.8)	34 (28.8)	–	144 (26.6)	
5 pads and more	1 (2.1)	4 (1.1)	1 (0.8)	–	6 (1.1)	
Total	47	375	118	–	541	
Any problem related to the sanitary materials						
No	26 (55.3)	304 (80.9)	63 (53.4)	80 (98.9)	473 (76.1)	Chi2 = 74.5548, Pr = 0.000
Yes	21 (44.7)	72 (19.1)	55 (46.6)	1 (1.1)	149 (23.9)	
Total	47	376	118	81	622	
Problems related to sanitary materials						
Itching	18 (85.7)	44 (63.8)	40 (72.7)	–	102 (70.4)	Chi2 = 4.7, Pr =0.3
Skin rashes	2 (9.5)	11 (15.9)	5 (9.1)	–	18 (12.4)	
Irritation	1 (4.8)	14 (20.3)	10 (18.2)	–	25 (17.2)	
Total	21	69	55	–	145	
Occupation						
School students	43 (91.5)	47 (12.5)	115 (97.5)	1 (1.2)	206 (33.1)	chi2 (9) = 555.3492 Pr = 0.000
College students	4 (8.5)	198 (52.7)	3 (2.5)	1 (1.2)	206 (33.1)	
Homemaker	0	116 (30.9)	0	79 (97.6)	195 (31.3)	
Job or Working	0	15 (3.9)	0	0	15 (2.5)	
Total	47	376	118	81	622	
Average pad requirement per cycle	10.93 ± 3.66	9.85 ± 3.75	11.94 ± 3.77	10.40 ± 3.84		Average pad requirement per cycle
Average cost incurred per cycle (Rs.)	26.67 ± 5.77	48.91 ± 24.72	46.13 ± 22.50	48.12 ± 24.20		Average cost incurred per cycle (Rs.)

### 3.5 Problems related to use of sanitary materials

**Figure 4:**
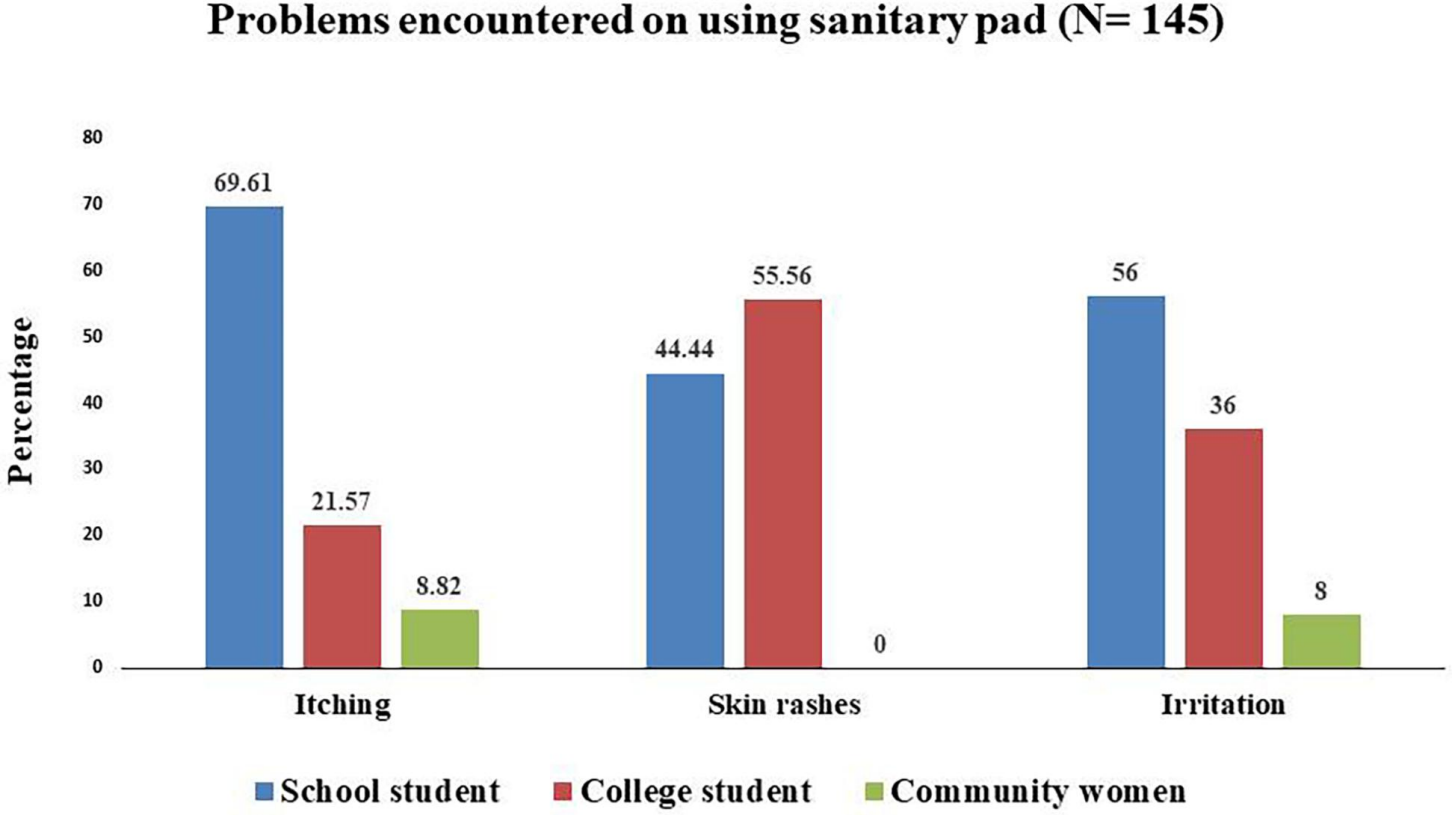
Health-related problems during menstruation.

Figure 4 illustrates the distribution of health-related issues associated with the use of sanitary pads among school students, college students, and community women. Among the 149 (23.9%) sanitary pad users who reported pad-associated problems, we only calculated this for 145 participants who could define their problems properly. From them, 93 (64.14%) were school students, 41 (28.2%) were college students, and 11 (7.5%) were community women.

**Figure 5:**
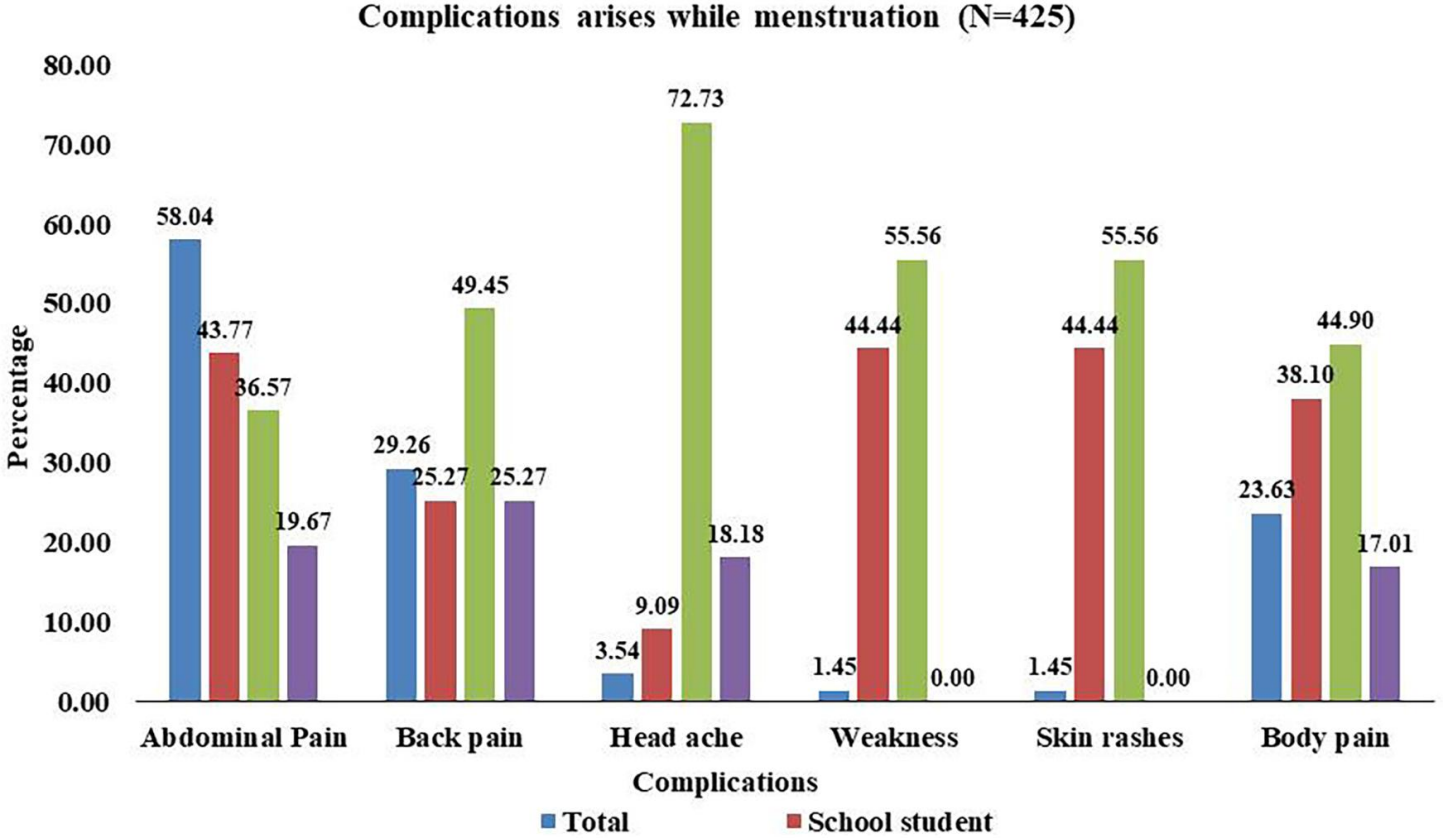
Health related problems encountered during menstruation cycle.

During the physiological process of the menstrual cycle, many females encounter different health-related problems. The reported problems in our study include abdominal pain (58.04%), back pain (29.26%), headache (3.54%), weakness (1.45%), skin rashes (1.45%), and body ache (23.66%). Upon comparing these health problems across the three categories of study participants, college students were found to encounter these problems more, followed by school students. The detailed distribution of different menstrual cycle-related health problems across the categories of participants is presented in Figure 5.

## 4 Discussion

Many women in rural India avoid discussing menstrual hygiene because it is still considered as a taboo in many places and communities. The current study attempted to understand how major different groups of reproductive-age women manage their menstrual hygiene in rural areas, so the findings could further be used to tailor interventions to specific needs. Along with this, it mainly focused on the menstrual materials, that they used during their menstruation, as it is suggested one of the 4 key indicators by the WHO/ UNICEF joint monitoring program for water, sanitation and hygiene (9). Our study revealed that a majority of participants chose sanitary pads (either purchased or government-supplied) as their preferred choice, particularly school & college students. Most of our study participants relied on self-procured compared to government-funded pads and both. None of the participants were found to use any other sanitary products such as menstrual cups, tampons, or discs. Most of the community women use clothes as their preferred absorbent. Most of the cloth users reported their experience as “excellent” compared to others, while most of the government-funded pad users rated their experience as “dissatisfied”. Furthermore, in disposal methods, it is revealed that burying pads is the most common method. Additionally, it was found that longer menstrual duration and menstrual irregularities were seen commonly among school students.

The present study revealed that a majority of participants preferred sanitary pads (either purchased or government-supplied), which aligns with the findings of a similar study among Indian women aged 18–30 years, where 94% of participants reported using sanitary pads (13). In contrast, a study conducted a few years ago among women of reproductive age found that only 57% used sanitary pads (11). This indicates a significant increase in the use of sanitary pads, even in rural areas, over time. This improvement could be attributed to the implementation of several menstrual hygiene programs in rural regions. For example, the Khusi program distributes a pack of six sanitary napkins, and the Rashtriya Kishor Swasthya Karyakram (RKSK) initiative in Odisha supports adolescent well-being by enabling Accredited Social Health Activists (ASHAs) to provide affordable sanitary pads to rural girls (14).

We also found that college students primarily used sanitary pads they purchased themselves, consistent with findings from a study in Delhi (15). We found that among school students the pad users are 100% which is lso consistent with the study that reported 100% of the school students are using pads (16). Among community women 62.38% of community women were using sanitary pads which is more than a previous reported study that reavealed around 39.8% of community womens were using sanitary pads (17). However, none of the community women in our study reported using pads from the Khusi program. Instead, most continued to use cloth to manage their menstrual flow. Although the Khusi program supplies subsidized sanitary pads to rural women through ASHA workers at a cost of INR 6 for six pads (18), our results suggest that these products were either unavailable or the women were unaware of the program. This discrepancy highlights the need for further research to explore gaps in the supply chain and awareness. Strengthening the distribution system and improving outreach efforts could enhance access and encourage sanitary pad usage among rural women.

Upon analyzing the levels of satisfaction with different types of menstrual materials, it was observed that most government sanitary pad users reported dissatisfaction. This was followed by those who used a combination of government-supplied and self-procured pads. Notably, participants who used cloth as an absorbent often rated their experience as excellent. This discrepancy in satisfaction levels may be attributed to factors such as the lower absorption capacity, unsuitable size and shape, and prolonged wet contact surface associated with some sanitary materials—particularly government-supplied pads. These issues can contribute to discomfort and dissatisfaction.

In addition to satisfaction levels, some health concerns were reported by sanitary pad users, with more complaints emerging from users of government-provided pads than those using self-procured ones. Commonly reported problems included itching and skin rashes, especially among school-going students. This might be due to use of certain sanitary pads containing organic solvents that affect the skin and cause allergic reaction resulting in skin rashes and itching (19). Use of sanitary pads for a prolonged duration as in schools could also be a reason for this. Nonetheless, female students find it difficult to change sanitary pads and are forced to go for extended periods of time without changing pads due to the lack of adequate water, sanitation, and hygiene (WASH) facilities in schools. This may result in skin rashes and discomfort (19).

Subsidising sanitary napkins for adolescent girls is an innovative step that promotes menstrual hygiene and aids in the prevention of RTIs in India (20). A previous study conducted in Odisha revealed that inadequate menstrual hygiene may pose health risks, such as reproductive and urinary tract infections (21). So, It is necessary to have adequate separate rest rooms with water, washing and changing facility in every school and colleges for girl students. This will also reduce the absenteeism among female students during menstruation. It is evidenced that Menstrual material is one of the four indicators recommended by the WHO (World Health Organization)/UNICEF (United Nations International Children's Emergency Fund) joint monitoring program for water, sanitation, and hygiene monitoring. This indicator focused on the type of material used in sanitary pads and its safe disposal and menstrual experiences (22).

Our study noticed that majority of the participants (47.3%) dispose their used pads by throwing them outside, whilst only a small number of participants (12.38%) prefer to bury them underground. In a study conducted by Van Eijk AM et al. reported that approximately 23% dumped the absorbent material in open areas (15), another study found that approximately 64.6% of the respondents dispose their menstrual absorbents in the bush or field (16). These findings highlight the priority for having a proper disposal method and practice in place especially in rural areas. A probable reason for using unhygienic methods of disposal could be due to prevailing taboos surrounding menstruation and lak of awareness about proper disposal methods. Most of the time these products are hidden from male members of the family and women in rural areas do not go out of their homes due to socio-cultural barriers resulting in a lack of means for its safe disposal. Nonetheless, the disposal of used pads has become a major issue in India. This is primarily due to the lack of an efficient disposal system in a variety of community and institutional settings, including schools, colleges, workplaces, and hospitals (23). As a result, used sanitary pads end up in municipal sewer systems, landfills, rural fields, and bodies of water. So, innovative solutions need to be developed and implemented to manage the menstrual waste materials.

Furthermore, we found the average cost of self-purchased sanitary pads (INR 48.91 ± 24.72) was higher than that of government-funded and combined users. This puts an extra financial burden on the females especially in rural areas. Considering the fact that commercial sanitary pads are non-reusable, expensive, and not environment friendly, other method like menstrual cup could be a better option for women in rural settings to promote their menstrual hygiene management (24).

Our study also noticed that college students had a higher prevalence of experiencing health problems during menstruation like headaches and weakness, whereas abdominal pain found more in school students which can be attributed to a variety of factors. Apart from that, menstruation irregularity was noticed more among school students. Due to academic and social pressures, students frequently experience high levels of stress. Stress can aggravate menstrual symptoms, resulting in health problems. Irregular sleep patterns, dietary habits, and exercise routines can all disrupt hormonal balance, making students more prone to menstrual irregularity and discomfort (25, 26). Social support system in families, peer counselling and seeking health care from professionals would help to address these problems.

### 4.1 Policy implication

The Govt. of India has launched initiatives through National Health Mission (NHM) to improve awareness about menstrual hygiene among young women and encourage the use of sanitary practices during menstruation. To improve accessibility and affordability, accredited social health activists (ASHAs) in rural areas are required to sensitize and distribute NHM's subsidized sanitary napkin. However, sensitization is required about the pros and consequences of menstrual hygiene management. The matter should be discussed openly in schools, colleges, and in community level. Results also showed an urgent necessity for a shift of the practices towards reusable material such as menstrual cups. In 2020, the Central Government launched ’Suvidha', a brand of 100% biodegradable sanitary napkins that were available at a discounted price in government-run Jan Aushadhi Kendras. However, access to and affordability of hygienic methods in rural areas remains a concern even today. So, a collaborative effort involving non-governmental organizations (NGOs) and local businesses hub is required for an establishment to facilitate the distribution of affordable and environmentally friendly menstrual products in rural communities.

### 4.2 Strengths and limitations

Our study attempts to investigate a sensitive and less researched topic among women of reproductive age group in rural communities among three different groups of participants. Most of the evidence on menstrual hygiene practices predominantly focus either on adolescent girls or among urban areas. We employed a random sample that broadens the generalisability of the findings. However, information on participants’ knowledge, cultural beliefs, and cleanliness practices are limited in this study. There may be selection bias due to participants being from divert settings that would potentially limit the generalizability of the findings.

## 5 Conclusion

Most participants in the study used sanitary pads compared to reusable absorbent materials, although, there was a lack of standard disposal methods. Policy reform by introducing comprehensive awareness program by way of community engagement could support improved disposable approaches, including the reduction of carbon emissions. Considerations to use biodegradable, and improved absorbent materials should be considered by manufacturers. Government partnerships with NGOs to implement village-level menstrual waste management systems, subsidies for eco-friendly pad production, and mandatory disposal education in school health programs could mitigate the problem.

## Data Availability

The original contributions presented in the study are included in the article/Supplementary Material, further inquiries can be directed to the corresponding author.
