# Supplementary material for: Menstrual hygiene management practices among rural females: findings from a rural health demographic environmental surveillance system (HDESS) cohort in Odisha, Eastern India
Source: Front Glob Womens Health. 2025 Oct 13;6:1617970. doi: 10.3389/fgwh.2025.1617970 (PMC12554699; doi:10.3389/fgwh.2025.1617970)
Supplement: Supplementary file 1 [file Table1.docx]

**Supplementary table 1:** The differences in cost and requirement of pads.

|  | **Usage of different commercial pad (n=316)** | | | | | |
| --- | --- | --- | --- | --- | --- | --- |
| Satisfaction level | Comfy (n=173) | Niine (n=16) | Whisper (n=59) | Stayfree (n=221) | Mix brand (n=17) | |
| Satisfied | 35 (29.66) | 3 (21.43) | 6 (19.35) | 57 (40.43) | | 2 (16.67) |
| Dissatisfied | 83 (70.34) | 11 (78.57) | 25 (80.65) | 84 (59.57) | 10 (83.33) | |
| Any complication due to use of pad |  |  |  |  |  | |
| No | 90 (76.27) | 12 (85.71) | 25 (80.65) | 119 (84.40) | 8 (66.67) | |
| Yes | 28 (23.73) | 2 (14.29) | 6 (19.35) | 22 (15.60) | 4 (33.33) | |
| Profession |  |  |  |  |  | |
| School student | 14 (11.86) | 0 | 7 (22.58) | 16 (11.35) | 3 (25.00) | |
| College student | 94 (79.66) | 12 (85.71) | 18 (58.06) | 61 (43.26) | 8 (66.67) | |
| Community women | 10 (8.47) | 2 (14.29) | 2 (19.35) | 64 (45.39) | 1 (8.33) | |
| Average pad requirement per cycle | 9 ± 3 | 10 ± 2 | 9 ± 3 | 10 ± 3 | 12 ± 5 | |
| Average cost incurred per cycle (Rs) | 47 ± 21 | 64 ± 23 | 47 ± 19 | 52 ± 29 | 54 ± 18 | |
